# Excelsior of Agriculture

**Published:** 1855-11

**Authors:** 


### EXCELSIOR OF AGRICULTURE.

A LARGER proportion of farmers fill our madhouses than any other one class of persons in the land. We have before stated the reason of this to be, the monotony of their employment, and want of mental stimulus. But this state of things is rapidly changing, by the influence of *Agricultural Journals*, which are establishing themselves in every section of the country; their tendencies are of a healthful character in many ways. By telling the reason of things, they open up a new world of thought to the cultivator of the soil, which is pursued under the influence of a stimulus the most potent in all lands, that of profit. Just give the most ordinary farmer an inkling of how he may make one acre produce as much as an acre and a half did before, and he will dive into the subject with an avidity quite surprising. Then there is the *pleasure of intelligent cultivation*, which is not inferior in its effect on the whole man, to the satisfaction of increased profits, while it is far purer and more elevating.

Furthermore, the character of these journals, as a class, and I know of not a single exception, is solid, substantial, on the side of virtue, integrity and industry.

For variety, a few columns in each paper or magazine are devoted to subjects not strictly agricultural, but of a more domestic nature, embracing home comforts, family management, and social virtues; and when it is remembered that very many farmers' families have but few facilities or opportunities of access to what is called the current literature of the day, this becomes a no small item of domestic education. These publications being issued but once or twice a month, are afforded at a very low rate, in some instances as low as fifty cents a year, and being seldom received, are read over and over again by, perhaps, every member of the family, and thus all that is said incidentally of morals, virtue and religion, has time to take root and spring up to a valuable fruitage. The men who have the taste and talent for conducting agricultural journals are, almost of necessity, safe men, solid men, men who are on the side of religion, for the time they have spent in these pursuits practically, has not allowed them the leisure to have their minds poisoned by German transcendentalism, their morals perverted by boarding-house, club and Spa life, their passions inflamed by yellow-covered romances and love stories, or the indecencies of George Sand, Mary Lyndon and the Escaped Nun, nor, indeed, have they had hours to spare to run after fashionable preachers and lecturers, to whom the crowd give the "all hail" in proportion as they ignore "the old paths," and pay court to "literal views," "freedom of thought," "rationalistic ideas," "progress," and all that. Such being the character of too many lecturers and editors of weekly papers in our large cities. Agricultural labors and studies have, in our opinion, a truly religious influence on the mind and heart of those who engage in them, the more decided, in proportion as they are more intelligent. It has been beautifully said, that if there exists one on earth who can eat his bread in peace with God and man,—it is he who has brought that bread out of the ground, for it is cankered by no fraud, it is wet by no tears, it is stained by no blood.

The *Ohio Cultivator*, at Columbus, published twice a month, for a dollar a year, has, in the September number, besides its regular agricultural items, such articles as "A Happy Childhood," "Propriety of Dress," "The Lady Equestrian." In any number of "*The Ohio Farmer*," at Cleveland, we have a large amount of reading on scientific and domestic subjects, and which may well afford a whole family full materials for a week's profitable reflection and conversation; and of a more varied character, still as safe are "*The American Agriculturist,*" of New York, "*The Country Gentleman,*" of Albany, and the "*Philadelphia Horticulturist.*" Let every farmer in the land take one of these publications.

